# Consistently Low Prevalence of Syphilis among Female Sex Workers in Jinan, China: Findings from Two Consecutive Respondent Driven Sampling Surveys

**DOI:** 10.1371/journal.pone.0034085

**Published:** 2012-04-23

**Authors:** Meizhen Liao, Xijuan Nie, Rongjian Pan, Chuangxin Wang, Shiman Ruan, Changqing Zhang, Dianming Kang, Jihua Fu, Yuesheng Qian, Xiaorun Tao, Jinkou Zhao

**Affiliations:** 1 Institute for AIDS/STD Control and Prevention, Shandong Provincial Center for Disease Control and Prevention, Jinan, Shandong Province, China; 2 Institute for AIDS/STD Control and Prevention, Licheng District Center for Disease Control and Prevention, Jinan, Shandong Province, China; 3 Institute for AIDS/STD Control and Prevention, Jinan Municipal Center for Disease Control and Prevention, Jinan, Shandong Province, China; 4 Monitoring and Evaluation Unit, The Global Fund to Fight AIDS, Tuberculosis and Malaria, Geneva, Switzerland; Asociacion Civil Impacta Salud y Educacion, Peru

## Abstract

**Background:**

Routine surveillance using convenient sampling found low prevalence of HIV and syphilis among female sex workers in China. Two consecutive surveys using respondent driven sampling were conducted in 2008 and 2009 to examine the prevalence of HIV and syphilis among female sex workers in Jinan, China.

**Methods:**

A face-to-face interview was conducted to collect demographic, behavioral and service utilization information using a structured questionnaire. Blood samples were drawn for serological tests of HIV-1 antibody and syphilis antibody. Respondent Driven Sampling Analysis Tool was used to generate population level estimates.

**Results:**

In 2008 and in 2009, 363 and 432 subjects were recruited and surveyed respectively. Prevalence of syphilis was 2.8% in 2008 and 2.2% in 2009, while no HIV case was found in both years. Results are comparable to those from routine sentinel surveillance system in the city. Only 60.8% subjects in 2008 and 48.3% in 2009 reported a consistent condom use with clients during the past month. Over 50% subjects had not been covered by any HIV-related services in the past year, with only 15.6% subjects in 2008 and 13.1% in 2009 ever tested for HIV.

**Conclusions:**

Despite the low prevalence of syphilis and HIV, risk behaviors are common. Targeted interventions to promote the safe sex and utilization of existing intervention services are still needed to keep the epidemic from growing.

## Introduction

The dramatic social and economic transition over the past three decades in China has led to dramatic social structure changes with increasing economic disparities, individual freedom, and broader liberation of sexual attitudes. Heterosexual transmission has become a major mode of HIV/AIDS transmission in China. Of the estimated 780,000 infections in 2011, 46.5% contracted HIV through heterosexual contact, 28.4% through injection drug use, 17.4% through homosexual contact and 6.6% through blood transmission in China [1]. The proportion of heterosexual contact has also been increasing in the newly reported infections in Shandong, an eastern coastal province in mainland China, reached 45.1% in 2010 [2], [3]. The experience of African countries has already indicated that when the heterosexual transmission became the main mode of HIV transmission, the epidemic grew rapidly [4]. Of the contributors to the high proportion of heterosexual transmission, transmission from injection drug using population was precluded as injection drug use was never a problem in Shandong. Reportedly transgender from men to women was rare in Jinan, neither in China. One particular attention was given to men who have sex with men (MSM) as this population has been reportedly to have a high rate marriage, which could potentially bridge the MSM population with heterosexual network. While the HIV prevalence has been increasing from 0.05% in 2007 to 3.1% in 2008 based on two consecutive respondent driven sampling (RDS) surveys among MSM in Jinan, the capital city of Shandong Province [5], unprotected virginal sex has been high although declining from 2007 to 2008 [5], [6]. MSM may have played a role in the high proportion of heterosexual transmission as reported in Peru [7]. The role of married MSM and those having unprotected virginal sex might play in bridging HIV infection to their female partners may be more complex than simply being a function of being married and having female partners. More detailed research on the topic of married MSM in China [5], [6]. The last potential contributor to heterosexual transmission of HIV is through contact with female sex workers (FSW), which is of particular concern [8], [9]. FSW, as a special bridge group, may play an important role in the spread of HIV epidemic [10].

However, increasingly high proportion of heterosexual transmission among reported HIV/AIDS cases is not supported by consistently low prevalence of HIV among FSW through routine sentinel surveillance in Shandong province [2], [3], neither in China [11]. As routine sentinel surveillance adopted convenient sampling, representativeness of the samples is questionable, thus their results may not be generalizable [12], [13]. RDS is a method of chain-referral sampling for obtaining a more representative sample from a hidden population [14]. It has been well applied in recruiting hidden populations such as injection drug users (IDU), men who have sex with men (MSM) and FSW worldwide [15]–[17]. In China RDS has been used widely to recruit MSM [18], piloted to recruited FSW [19]. Those studies showed that RDS was a feasible sampling approach to recruiting hard-to-reach population.

Jinan, the capital of Shandong province, is known as the “Spring City” due to the large number of natural springs and lakes within its borders. Many of these springs and lakes have been made into parks and tourist attractions. Along with economic development, Jinan has gradually become the economic center of Shandong province, which ranks 2^nd^ in gross domestic product in China. Jinan has a total population of 6.7 million, small to moderate size by Chinese standards. Of Jinan’s total population, 5.9 million are official residents (holding Jinan hukou) and another 800,000 who live as migrants in the city. It is estimated that 50,000–100,000 employees work in entertainment establishments and about one-third of them are FSW (15,000 to 30,000) [20]. Heterosexual transmission accounted for 50% of Jinan’s estimated epidemic in 2009 [2], [3]. One sentinel site adopted convenient sampling for FSW has been established in Jinan since 2003, with 1 HIV positive (1/280) identified in 2004 and 2 (2/602) in 2005, and with a syphilis prevalence of 0.56% in 2004 and 1.49% in 2005 [2], [3]. To examine the prevalence of syphilis, HIV and risk behaviors, two consecutive surveys among samples of FSW obtained through RDS were conducted in 2008 and in 2009 in Jinan.

## Methods

### Overall Study Design and Sampling Method

The two surveys were conducted by the same group of trained health professional staff at a fixed interview site, located at Licheng District Center for Disease Control and Prevention (CDC) in Jinan, China. The sampling of participants strictly followed pre-established procedures based on RDS theory. Two consecutive surveys used the exactly same questionnaire. In brief, RDS begins with a formative assessment research, including in-depth interviews with stakeholders and informal meetings with mammies, which helped gathering basic information about FSW in Jinan, the way of accessing them, and selection of candidates of first group of subjects, i.e., seeds, type and level of incentives, information to be collected in the questionnaire. Eligibility for the surveys was defined as ‘a female aged 18 years old or above who has exchanged sex for money or goods in the past month, and has been living in Jinan for at least 6 months’ when the surveys started. A few individuals identified during formative assessment research are invited to start the recruitment process. These initial individuals, so-called seeds, after completing a behavioral survey and serological testing, were explained the eligibility and procedures, and were invited to recruit three other eligible FSW peers from their social networks to extend chains of recruitment. Seeds and their recruits were given a primary incentive package including 50 Chinese Yuan (approximately U.S. $ 7.3), HIV prevention pamphlets and four boxes of condoms for participation in the survey, plus an additional 20 Chinese Yuan (U.S. $ 2.9) for recruiting one additional peer as secondary incentives. In 2008 and in 2009, 7 and 4 seeds were selected representing diversity with respect to educational attainment, venue, income and marital status, respectively. As the recruitment chains extended, the key demographic variables, including age, residency status (hukou), marital status and education, and key behaviors such as condom use, were monitored to assess the equilibrium. The question used for the network size was ‘How many FSW can you identify in Jinan? You know their names or nicknames and contacted during the past month?’ The survey protocol was approved by the Institutional Review Board of Shandong Provincial CDC. A written informed consent was obtained from each subject, after explaining the potential risks and benefits of the survey, voluntary participation, ensured anonymity and confidentiality. To minimize the repetitive participation, specific features of the individual, such as wearing a glass, tattoo, etc, were recorded and checked during the registration prior to survey. The same staff was responsible for registration for both 2008 and 2009.

### Behavioral Measures

Questionnaire-based interviews provided information, including social network size, demographic information such as age, marital status, ethnicity, hukou, education, income, behavioral information such as sex behavior and condom use with regular, non-regular non-paying and commercial partners, types of drug use, ever injecting drug, self reported history of sexually transmitted diseases (STD), STD treatment, perceived personal risk for HIV infection, history of an HIV test, and HIV-related prevention services received in the past 12 months. The use of HIV-related prevention service was assessed by whether the subject has ever received any of the 5 relevant prevention services in the last 12 months, including free condoms, peer education, STD diagnosis and treatment, HIV counseling and testing, or free HIV related materials.

### Serologic Measurements

After questionnaire interview, an intravenous blood sample was collected from each of the participants for HIV-1 and syphilis tests. HIV-1 antibody was determined by two screening tests with different enzyme-linked immunoassay kits (Beijing Jinhao Biologic Production Co. and Beijing Wantai Biological Medicine Company, China). If both were negative, the result was recorded as HIV negative. If both were positive or there was discordance in the results, the specimen was sent for confirmation using Western Blot (HIV Blot 2.2 WB; Genelabs Diagnostics, Singapore). If Western Blot is positive, the subject is classified as HIV positive. If Western Blot is negative or undetermined, the subject is followed up every 3 months to 6 months according to China National Guideline for the Detection of HIV/AIDS [21]. Syphilis infection was determined using rapid plasma reagin (RPR, Beijing Wantai Biological Production Company, Beijing, China) and a Passive Particle Agglutination Test for Detection of Antibodies to *Treponema pallidum* (TPPA, Rong Sheng Biostix Inc, Shanghai, China). Syphilis infection was defined when both RPR and TPPA tests are positive. All participants were provided post-test and post-diagnosis counseling, and all syphilis cases were referred to the designated STD clinic for syphilis treatment, free of charge.

### Data Analysis

Questionnaire-based data and biological results were double checked for logic consistence and recorded into the EpiData software (EpiData 3.0 for Windows). Unprotected sex was defined as not using condoms 100% of the time for all the sex acts in the past month, including any type of sex (oral, vaginal, or anal sex) with any kind of partner (clients, regular non-paying, or non-regular non-paying partners). Self-reported history of STD in last year was defined as ever had pain or burning sensation when urinate or genital ulcer or hyperplasia. Respondent Driven Sampling Analysis Tool (RDSAT version 6.0.1) was used to produce estimates of the population level prevalence of each factor and their confidence interval. The statistical significance was determined by examining if there are overlaps in confidence intervals of variables between in 2008 and in 2009.

## Results

The two surveys were conducted from February to August in 2008 and from May to October in 2009. Seven seeds were selected in 2008, with their marital status of 1 single, 3 married, 2 separated and 1 widowed; education of 2 primary school, 2 middle school and 3 high school; venue of 2 KTV, 2 sauna, 2 teahouse, and 1 non-venue based. Four seeds were selected in 2009, with their marital status of 1 single, 1 married, 1 separated and 1 widowed; education of 1 primary school and 3 high school; venue of 1 KTV, 1 sauna, 1 teahouse and 1 non-venue based. Among 1,022 and 1,092 coupons distributed in 2008 and in 2009, 35.5% and 39.1% returned, which resulted in 363 and 432 subjects recruited in 2008 and in 2009 respectively. Equilibrium were reached on the key variables tracked during recruitment (age, hukou, marital status, education, income) after 13–21 waves in 2008 with the longest chain reaching 25 waves and 11–18 waves in 2009 with the longest chain reaching 21 waves. Of all participants in 2008 and in 2009 73 subjects reported participating in both surveys.

### Characteristics of Participants

The compositions of samples in 2008 and in 2009 were not different by age, ethnicity, education and recruited venues. The proportion of subjects aged above 25 years was 52.2% in 2008 and 63.0% in 2009; Most of the participants were Han Chinese with 90.2% in 2008 and 95.9% in 2009; over 85% received middle school or higher education with 37.5% in 2008 and 35.9% in 2009 received high school or higher education. The compositions of samples by marital status, hukou and income were different in 2008 and in 2009. Being single never-married accounted for 37.5% (95% CI: 30.9–44.0) in 2008 and 27.6% (95% CI: 22.4–33.6) in 2009 while the proportion of being separated or widowed increased from 20.9% (95% CI: 16.9–25.9) in 2008 to 31.8% (95% CI: 26.6–36.8). Having Jinan hukou increased from 2008 (4.3%; 95% CI: 2.2–6.5) to 2009 (16.3%; 95% CI: 11.8–21.4) while the proportion of subjects coming from other provinces decreased from 70.2% (95% CI: 64.3–76.2) in 2008 to 48.2% (95% CI: 42.2–54.2) in 2009. The proportion reporting monthly income ≥5,000 Chinese Yuan (U.S. $ 730) decreased from 41.0% (95% CI: 35.6–48.5) in 2008 to 12.7% (95% CI: 8.5–18.0) in 2009 (Table 1).

**Table 1:** Crude and population-adjusted demographic characteristics among female sex workers, Jinan, China from 2008 to 2009.

Variable	2008 (N = 363)		2009 (N = 432)	
	Crude	Adjusted	Crude	Adjusted
	% (N)	% (95% CI)	% (N)	% (95% CI)
**Age**				
≤20	12.7 (46)	16.0 (10.7–21.0)	9.7 (42)	11.6 (7.9–15.7)
21∼	29.8 (108)	31.8 (25.7–36.4)	23.8 (103)	25.4 (20.6–30.7)
25∼	28.1 (102)	25.0 (20.1–29.4)	26.4 (114)	24.5 (20.0–29.6)
30∼	29.5 (107)	27.2 (23.8–33.9)	40.0 (173)	38.5 (31.7–44.6)
**Ethnicity**				
Han	92.8 (337)	90.2 (86.0–94.4)	95.8 (414)	95.9 (93.9–97.5)
Non-Han minority	7.2 (26)	9.8 (5.6–14.0)	4.2 (18)	4.1 (2.5–6.1)
**Education**				
Primary school or lower	8.5 (31)	7.7 (5.0–10.6)	13.0 (56)	13.2 (9.8–17.1)
Middle school	55.4 (201)	54.8 (48.9–60.9)	52.8 (228)	50.9 (45.9–55.8)
High school or higher	36.1 (131)	37.5 (31.4–43.5)	34.2 (148)	35.9 (30.9–40.8)
**Marital status**				
Single	34.3 (124)	37.5 (30.9–44.0)	24.5 (106)	27.6 (22.4–33.6)
Cohabited	21.0 (76)	22.4 (16.8–27.1)	16.0 (69)	15.5 (11.6–19.6)
Married	23.5 (85)	19.1 (15.2–24.3)	27.1 (117)	25.0 (20.8–29.4)
Separated or widowed	21.3 (77)	20.9 (16.9–25.9)	32.4 (140)	31.8 (26.6–36.8)
**Residency**				
Jinan city	5.5 (20)	4.3 (2.2–6.5)	13.0 (56)	16.3 (11.8–21.4)
Shandong not Jinan	24.0 (87)	25.6 (20.0–31.0)	33.8 (146)	35.5 (30.1–40.6)
Non-Shandong Province	70.5 (256)	70.2 (64.3–76.2)	53.2 (230)	48.2 (42.2–54.2)
**Venues**				
Sauna/bath	17.1 (62)	16.6 (7.9–27.9)	6.7 (29)	6.1 (2.2–20.6)
KTV, bars	42.1 (153)	36.6 (28.4–45.2)	40.5 (175)	36.4 (29.9–42.8)
Tea house/hotel	32.2 (117 )	31.9 (23.9–39.3)	34.0 (147)	32.4 (27.3–38.7)
Hair beauty salons/foot massage	5.0 (18)	8.0 (4.0–12.0)	9.3 (40)	12.2 (7.7–16.5)
Non-venue based	3.6 (13)	7.0 (2.8–11.5)	9.4 (41)	12.9 (7.6–19.1)
**Income (RMB)**				
<2000	20.0 ( 72)	26.0 (18.5–30.6)	17.4 (75)	20.7 (16.4–25.0)
2000∼	35.0 (126)	33.0 (27.8–39.5)	69.7 (301)	66.6 (61.0–71.4)
≥5000	45.0 (162)	41.0 (35.6–48.5)	13.0 (56)	12.7 (8.5–18.0)

### HIV/Syphilis Prevalence and Sexual Behaviors

No HIV positive was found in 2008 and in 2009; Syphilis prevalence were 2.8% (95% CI: 1.2–5.6) in 2008 and 2.2% (95% CI: 1.0–3.7) in 2009. The sex debut ranged from 10 to 30 years old, with a mean of 20.3 years old in 2008 and 20.2 in 2009. The age at the first commercial sex ranged from 18 to 47 years old with the mean age was 26.2 years old in 2008 and 26.6 in 2009. The proportion of being ≥25 years old at the first commercial sex increased from 36.0% (95% CI: 30.7–42.9) in 2008 to 49.7% (95% CI: 43.8–55.2) in 2009.The proportion of subjects with 14 or more clients in the past month accounted for 38.3% (95% CI: 30.2–47.4) in 2008 and 25.5% (95% CI: 20.3–30.7) in 2009 (Table 2).

**Table 2:** Crude and adjusted syphilis prevalence and risk behavior indicators among female sex workers, Jinan, China from 2008 to 2009.

Variable	2008		2009	
	Crude	Adjusted	Crude	Adjusted
	% (N)	% (95% CI)	% (N)	% (95% CI)
**Syphilis**				
Positive	3.1 (11)	2.8 (1.2–5.6)	2.6 (11)	2.2 (1.0–3.7)
Negative	96.9 (339)	97.2 (94.4–98.8)	97.4 (419)	97.8 (96.3–99.0)
**Age at first sex (years of age;** 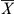 **± SD)**	20.3 ± 3.0		20.2 ± 2.6	
<20	42.1 (153)	40.8 (34.5–44.6)	41.0 (177)	39.8 (35.2–44.3)
≥20	57.9 (210)	59.2 (55.4–65.5)	59.0 (255)	60.2 (55.7–64.8)
**Age at first commercial sex (years of age;** 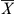 **± SD)**	26.2 ± 5.8		26.6 ± 6.5	
<20	20.4 (74)	24.1 (17.9–30.6)	20.8 (90)	22.4 (18.2–27.4)
20∼	38.0 (138)	40.0 (33.7–45.0)	28.2 (122)	27.9 (23.2–32.6)
25∼	41.6 (151)	36.0 (30.7–42.9)	50.9 (220)	49.7 (43.8–55.2)
**Number of clients in the past month**				
<7	46.0 (165)	45.2 (35.7–53.4)	55.1 (237)	53.4 (48.0–59.0)
7∼	18.7 (67)	16.5 (12.7–21.2)	20.0 (86)	21.1 (17.0–25.3)
14∼	35.4 (127)	38.3 (30.2–47.4)	24.9 (107)	25.5 (20.3–30.7)
**Condom use in the last episode with clients**				
Yes	89.8 (326)	88.5 (81.7–92.9)	87.7 (379)	86.3 (83.3–88.8)
No	10.2 (37)	11.5 (7.1–18.3)	12.3 (53)	13.7 (8.2–16.7)
**Condom use in the last month with clients**				
Never	0.8 (3)	2.7 (0.1–6.5)	2.1 (9)	2.9 (1.7–4.2)
Sometimes	12.4 (45)	12.3 (9.0–16.7)	8.8 (38)	8.8 (6.7–11.4)
Often	24.8 (90)	24.2 (19.8–29.1)	40.3 (174)	40.0 (35.2–45.1)
Always	62.0 (225)	60.8 (54.3–66.1)	48.8 (211)	48.3 (42.6–53.4)
**Ever had oral sex in the last month**				
Yes	38.0 (138)	39.1 (33.2–47.2)	27.5 (119)	28.6 (23.8–33.6)
No	62.0 (225)	60.9 (52.8–66.8)	72.5 (313)	71.4 (66.4–76.2)
**Condom use in the last month with clients when having oral sex with clients**				
Never	18.8 (26)	17.5 (10.2–28.8)	9.3 (11)	7.4 (3.0–15.9)
Sometimes	33.3 (46)	36.6 (25.1–52.9)	22.0 (26)	18.2 (8.1–29.1)
Often	14.5 (20)	10.0 (5.0–18.0)	19.5 (23)	25.0 (11.1–42.2)
Always	33.3 (46)	35.9 (18.3–44.7)	49.5 (58)	49.4 (32.3–63.2)
**Ever had anal sex in the last month**				
Yes	10.7 (39)	11.8 (8.1–16.9)	5.3 (23)	5.8 (3.6–8.5)
No	89.3 (324)	88.2 (83.1–91.9)	94.7 (409)	94.2 (91.5–96.4)
**Condom use in the last month when having anal sex with clients**				
Never	25.6 (10)	–	17.4 (4)	–
Sometimes	23.1 (9)	–	30.4 (7)	–
Often	15.4 (6)	–	0	–
Always	35.9 (14)	–	52.2 (12)	–
**Do you have non-regular non-paying partners**				
Yes	31.1 (113)	29.8 (24.6–35.4)	53.3 (229)	50.5 (45.3–55.8)
No	68.9 (250)	70.2 (64.6–75.4)	46.7 (201)	49.5 (44.2–54.7)
**Condom use with non-regular non-paying partners in the last episode**				
Yes	60.4 (67)	46.3 (19.3–65.4)	65.1 (149)	71.5 (61.5–78.8)
No	39.6 (44)	53.7 (34.6–80.8)	34.9 (80)	28.5 (21.3–38.5)
**Condom use with non-regular non-paying partners in the last month**				
Never	16.4 (18)	7.9 (0.9–19.9)	17.7 (41)	10.1 (4.7–16.1)
Sometimes	36.4 (40)	31.9 (13.6–50.0)	11.3 (26)	9.4 (5.6–14.1)
Often	12.7 (14)	21.9 (20.0–25.2)	39.0 (90)	48.5 (36.8–57.2)
Always	34.5 (38)	38.3 (16.4–60.9)	32.0 (74)	32.0 (24.1–43.7)
**Do you have regular partner of boyfriend**				
Yes	75.2 (273)	68.9 (64.1–75.5)	80.8 (349)	79.9 (75.7–84.5)
No	24.8 (90)	31.1 (24.6–35.9)	19.2 (83)	20.1 (15.5–24.3)
**Condom use in the last episode with regular sex partners**				
Yes	41.2 (112)	43.6 (34.7–51.5)	49.6 (174)	52.5 (46.2–57.7)
No	58.8 (160)	56.4 (48.5–65.3)	50.4 (177)	47.5 (42.3–53.9)
**Condom use in the last month with regular sex partners**				
Never	31.8 (85)	23.8 (17.8–29.6)	26.3 (92)	25.1 (19.8–30.3)
Sometimes	37.8 (101)	41.5 (35.3–51.4)	11.7 (41)	9.6 (6.8–12.7)
Often	11.6 (31)	12.2 (7.0–16.7)	38.6 (135)	38.5 (33.1–45.1)
Always	18.7 (50)	22.4 (13.8–29.8)	23.4 (82)	26.9 (21.2–31.7)
**Ever drinking alcohol**				
Yes	75.5 (274)	73.2 (66.7–79.6)	78.4 (338)	77.5 (72.6–81.9)
No	24.5 (89)	26.8 (20.4–33.3)	21.6 (93)	22.5 (18.1–27.4)
**Ever smoking**				
Often	34.3 (124)	32.8 (28.0–38.7)	23.7 (102)	23.4 (19.4–27.0)
Sometimes	21.3 (77)	20.6 (15.9–25.0)	24.4 (105)	25.5 (21.2–29.7)
Never	44.3 (160)	46.6 (41.0–51.9)	52.0 (224)	51.1 (46.5–56.4)
**Ever used illicit drugs**				
Yes	12.7 (46)	12.0 (7.8–14.9)	13.9 (60)	14.0 (11.1–17.0)
No	87.3 (317)	88.0 (85.1–92.2)	86.1 (371)	86.0 (83.0–88.9)
**Ever injecting drug**				
Yes	8.7 (4)	–	8.3 (5)	–
No	91.3 (41)	–	91.7 (55)	–
**Ever received HIV–related services in the last year**				
Yes	44.9 (163)	45.1 (38.9–51.7)	40.5 (175)	37.4 (32.2–43.0)
No	55.1 (200)	54.9 (48.6–61.2)	59.5 (257)	62.6 (57.0–67.8)
**Ever tested for HIV**				
Yes	18.7 (68)	15.6 (11.5–19.9)	19.4 (84)	13.1 (9.8–16.9)
No	81.3 (295)	84.4 (80.1–88.5)	80.6 (348)	86.9 (83.1–90.2)
**Self–reported history of STD in last year**				
Yes	19.0 (69)	17.3 (13.8–21.6)	25.2 (109)	24.9 (20.6–28.9)
No	81.0 (294)	82.7 (78.4–86.2)	74.8 (323)	75.1 (71.1–79.4)
**Medical service seeking behaviors**				
Sought public medical treatment	76.5 (273)	76.3 (71.3–81.2)	77.8 (336)	76.3 (72.0–80.5)
Sought private medical treatment	5.9 (21)	6.5 (4.0–9.4)	6.0 (26)	7.0 (4.4–9.7)
Self–treated STD symptoms	11.8 (42)	13.2 (9.3–17.3)	12.5 (54)	12.4 (9.3–15.8)
No treatment	5.9 (21)	4.0 (2.2–5.8)	3.7 (16)	4.3 (2.4–6.4)
**Will stop commercial sex when feel abdomen uncomfortable**				
Stop	81.5 (296)	84.2 (80.4–88.3)	92.1 (398)	93.2 (90.3–95.6)
Non-stop	18.5 (67)	15.8 (11.7–19.6)	7.9 (34)	6.8 (4.4–9.7)
**Regular antibiotic use to prevent STD infection**				
Yes	49.6 (180)	47.9 (43.3–54.0)	47.6 (205)	46.0 (40.3–51.4)
No	50.4 (183)	52.1 (46.0–56.7)	52.4 (226)	54.0 (48.6–59.7)

### Condom use with Sex Partners

Table 2 presents condom use with paying clients, non-paying non-regular partners and regular partners. In the last month 39.1% of subjects in 2008 and 28.6% in 2009 ever had oral sex with clients and 11.8% in 2008 and 5.8% in 2009 ever had anal sex behaviors. The proportions of consistent condom use in the last month when having vaginal or oral sex with clients were 60.8% or 35.9% in 2008 and 48.3% or 49.4% in 2009, respectively.

Increased proportion of FSW had non-regular non-paying partners or regular partners, from 29.8% or 68.9% in 2008 to 50.5% or 79.9% in 2009, respectively. Consistent condom use in the last month with non-regular non-paying partners or regular partners was 38.3% or 22.4% in 2008 and 32.0% or 26.9% in 2009.

The condom use in 2008 and 2009 were not different with all kinds of sex partners except a decreased consistent condom use when having vaginal sex with clients from 60.8% in 2008 to 48.3% in 2009.

### Drinking, Smoking and Drug use Behaviors

Most of subjects in 2008 (73.2%) and in 2009 (77.5%) drank alcohol during the past month, and half (53.4% in 2008, 58.9% in 2009) of them smoked. Over 10% subjects (12.0% in 2008, 14.0% in 2009) ever used illicit drugs, among which over 90% are club drugs (Table 2).

### Medical Service Seeking Behaviors and HIV-related Service Utilization

About 20% (17.3% in 2008, 24.9% in 2009) self reported a history of any STD symptoms or signs, among them about three quarters (76.3% in 2008, 76.3% in 2009) sought public medical services. When having uncomfortable abdominal pain, 15.8% did not stop commercial sex in 2008, which decreased to 6.8% in 2009. To prevent STD infection, 47.9% of subjects in 2008 and 46.0% in 2009 regularly used antibiotics (Table 2).

During the past year, 54.9% subjects in 2008 and 62.6% in 2009 have never received HIV-related services in the last year. Only 15.6% subjects in 2008 and 13.1% in 2009 have ever tested for HIV.

## Discussion

Two consecutive RDS surveys confirmed a consistently low prevalence of HIV and syphilis among FSW in Jinan, China. The low HIV and syphilis prevalence found in Jinan is similar to the results from routine sentinel surveillance among FSW in Jinan [2], [3], and the results from other second tier cities in China [19]. From 2004 to 2008, findings from 15 national HIV comprehensive surveillance sites for FSW indicated a median of syphilis prevalence from 0.5% to 1.8% [22]. Those results suggest that the HIV epidemic among FSW is not the major contribution to heterosexual transmission in Jinan.

Another study in Shandong province looked into the detailed categorization of reported HIV/AIDS cases and found a total of 312 HIV positive migrant women from high risk provinces were reported by the end of 2006, which accounted for 24.2% of all cases and 56.8% of female cases in the province [23]. The imported female cases in the province present a possible driver for the increasing proportion of heterosexual transmission, as most of them got married to local unmarried men. Active case identification and prevention is required for migrants to prevent the secondary transmission to male population. Although the findings in this study cannot be generalized to other provinces, further studies looking into other sources of infection than FSW are still needed. Given the increasing HIV prevalence among MSM in Jinan and in many other Chinese cities [5], [24], and high unprotected virginal sex between MSM and their female sex partners [5], [6], well designed studies are needed to examine possible spread of HIV from MSM to heterosexual network.

Despite the low HIV/syphilis prevalence found in Jinan, high unprotected sexual behaviors, such as low condom use, high number and frequent turnover of paying partners, together with high rates of drug use and self-reported STD-related symptoms, suggest a potential for the spread of HIV/STD between FSW and clients. Condom use can effectively prevent the transmission of HIV/STD, but only with a large enough coverage, as suggested by the Asian Epidemic Model [25], [26]. A low rate of HIV service utilization, a high rate of self STD treatment with antibiotics put FSW at an elevated risk of HIV/STD infection. Over 50% subjects have not been covered by any HIV-related services in the last year. A low rate of HIV test shall be of particular attention. Studies have shown the importance of HIV counseling and testing in engaging at-risk groups in intervention, care and treatment and in reducing the incidence of STD transmission and increasing condom use [27], [28]. More than 10% illicit club drug use could add another layer of risk for the risk of HIV/STD infection in Jinan. Club drug use is increasing in many areas of the world [29] and is associated with unprotected sexual practices and HIV/STD infection [30], [31]. Our previous studies had showed club drug abuse may play more importance role on HIV/STD epidemic in Shandong [32], [33]. Luckily, despite the high levels of risk behavior, the prevalence of HIV and syphilis is low. A window of opportunity for preventing HIV epidemic from growing still exists. A targeted intervention, among FSW in general and subgroups with higher unprotected sex behavior in particular, shall be designed and implemented.

Compared with those in 2008, the increased proportion of being separated or widowed but the decreased proportion of consistent condom use with clients was found in 2009. This subgroup FSW was often not in a position to negotiate condom use [34], and therefore poses increased risk for HIV and syphilis infection and transmission. Higher proportion of subjects with local hukou found in 2009, may be attributable to social structure and value changes after China’s “open door” policy in the past 3 decades, where commercial sex becomes more tolerable [35]. These subgroups shall be focus in the overall strategy for HIV and syphilis prevention among FSW.

The surveys were not without challenges and limitations. The growth of referral chain was not fast as suggested in RDS theory [36]. The duration of two surveys was longer than planned 3 months. The effect of extended duration (about 6 months in both 2008 and 2009) on sample composition is hard to quantify [37]. Similar to other behavioral surveys, behavioral information was relied on self-reporting, recall bias may exist. The nature of sensitivity for those sensitive sex-related questions may have information bias. However, the same group of trained interviewers conducted two surveys at the same site using exactly same questionnaire. Although random errors may still exist, it may be safe to conclude the low prevalence of syphilis based on ensured consistencies in method and procedures.

In addition, RDS application usually has a higher running cost than convenient sampling. Sentinel surveillances using convenient sampling limited the generalization of the results to the population at large. The relative small sample size may affect the generalizability of the survey results. In our study, the equilibrium was reached in both surveys. According to the theory of RDS, the sample composition shall be similar to that of population [14]. There was no difference found between RDS surveys and routine sentinel surveillance. This may be attributable to a number of factors, a) real HIV prevalence is very low, which is irrelevant to sampling method or representativeness of the samples; b) both samples from RDS and routine sentinel surveillance skewed to the subgroup with low HIV prevalence. However the chance is minimal. RDS is not designed for routine sentinel surveillance in a setting like Jinan, China. However its application for periodic surveys will be critical in helping to understand the real HIV prevalence in the population from a difference angle.
